# Evaluation of Heavy Metal and Microbial Contamination in Green Tea and Herbal Tea Used for Weight Loss in the Palestinian Market

**DOI:** 10.1155/2020/7631562

**Published:** 2020-11-10

**Authors:** Murad N. Abualhasan, Nidal jaradat, Mohammed Hawash, Rama Khayat, Eman Khatatbeh, Malak Ehmidan, Munir Al-Atrash

**Affiliations:** ^1^Faculty of Medicine and Health Sciences, Department of Pharmacy, An-Najah National University, Nablus, State of Palestine; ^2^Jerusalem Pharmaceutical Company, Quality Control Department, Ramallah, State of Palestine

## Abstract

The use of green tea and herbal tea for weight loss is increasing worldwide owing to the rising rates of obesity. There are concerns about the safety and quality of these herbal products owing to their increased consumption worldwide. In this study, we evaluated randomly collected samples of green tea and herbal tea and tested them for heavy metal and microbial contamination. Eighteen samples of green tea or herbal tea of widely used brands in Palestine were tested for heavy metal and microbial contamination. The results showed that 7 of the samples had toxic heavy metals such as chromium (Cr) and lead (Pb), and their concentrations were above the allowable limits set by the World Health Organization (WHO). Moreover, 6 of the samples that were tested had microbial contamination with high total aerobic microbial count (TAMC) and total yeast and mold count (TYMC). This could be due to improper handling and storage conditions of these herbal products. This study is the first of its kind in Palestine, and its results are forewarning to all the responsible authorities, including the Ministry of Health (MoH), to take immediate corrective actions such as quality control testing, auditing, and registration of marketed tea products.

## 1. Introduction

The use of green tea and herbal tea for weight loss has been increasing worldwide, concomitantly with the rates of obesity [1, 2]. These products are easily accessible, trusted, easy to use, inexpensive, and thought to be safer compared to conventional medications. These factors led to an increased use of these types of teas [3, 4]. Globally, tea is the second most consumed drink after water [5]. Three billion kilograms of tea are produced each year worldwide [6]. Nearly 20%–22% of the total tea consumption is of green tea, primarily in China, Japan, Korea, Morocco, and few other countries in North Africa and the Middle East [5, 7, 8].

Green tea is “nonfermented” and obtained from *Camellia sinensis*, whereas other herbal teas used for weight loss are from several other tea plants, which are sometimes used in combination with green tea. These include *Pimpinella anisum*, *Mangifera indica*, *Garcinia gummi-gutta*, *Terminalia bellirica*, *Aloysia citrodora*, *Matricaria chamomilla*, *Centaurea cyanus*, *Urtica*, *Eucalyptus globulus*, *Plantago ovata*, *Laurus nobilis*, *Tilia europaea*, *Cymbopogon citratus*, *Mentha spicata*, *Rosa canina*, *Echinacea angustifolia*, *Terminalia chebula*, *Vaccinium oxycoccos*, *Cuminum cyminum*, *Zingiber officinale*, *Salvia officinalis*, *Cinnamomum verum*, *Foeniculum vulgare*, *Portulaca oleracea*, and *Senna italica* [5, 9].

Huge concerns about the safety and quality of herbal products have been rising. This is due to the rapid and widespread increase in the consumption of herbal products over the last few years [10]. The level of heavy metal contamination in herbal products is recommended to be minimal because these consequently add to the level that has already been consumed through daily diet [10]. The allowable limits of heavy metals depend on the nature adjusted according to regional or national variations [10]. The allowable limits of selected heavy metals in edible medicinal plants, according to international guidelines, are provided in Table 1.

Microbial contamination in herbal products may occur at any stage of product preparation, from planting and harvesting to packaging, distribution, and storage. The presence of microorganisms at certain levels may indicate poor quality of such products [11]. The allowable limits of microbial contamination vary according to the type of herb and its intended use [10]. The allowable limits of microbial contamination in herbal products recommended by the WHO are the total aerobic microbial count (TAMC) must not exceed 10^7^ CFU/g; the total yeast and mold count (TYMC) must not exceed 10^5^ CFU/g; and these products must be free from *Clostridium difficile*, *Salmonella*, and *Shigella*.

Currently, there is an inclination towards widely using different types of herbal products as complementary and alternative medicine in Palestine, owing to the biodiversity in this area [12]. Herbal products in the Palestinian market are regulated and controlled by the Ministry of Health (MoH) through the General Administration of Pharmacy by the Drug Registration Department. This department is responsible for the registration of imported herbal products and food supplements. The affiliated quality control department is responsible for quality testing and implementing the rules of Good Manufacturing Procedure (GMP) for medicinal herbs packed in Palestinian establishments. The laws and regulations regarding herbal products have been recently established and updated. The MoH has the right to withdraw any herbal products that prove to have a dangerous side effect. The recommendations of the MoH on the allowable limits of heavy metals in herbal medicine are based on the recommendations of the WHO and Food and Agriculture Organization (FAO) [13–15]. However, there is still a gap in implementing these rules and regulations with regular quality testing for microbial and heavy metal contamination in green tea and herbal tea that are sold in the Palestinian market [16].

Several studies were carried out globally to evaluate the contamination in herbal products. These were conducted in countries, such as Pakistan, Tanzania [17–20], Italy [21], Ghana [22], Nigeria [23], Brazil [24], South Africa [25] and some Arab countries, such as Palestine, Saudi Arabia, the United Arab Emirates, and Egypt [26–30]. The main objective of these studies was to detect the presence of microbial and/or heavy metal contamination, such as lead (Pb), cadmium (Cd), manganese (Mn), iron (Fe), chromium (Cr), zinc (Zn), mercury (Hg), nickel (Ni), and arsenic (As) in some common medicinal plants and types of teas and to assess their safety according to international limits.

The results of these studies showed that manganese and cobalt exceeded the allowable limits [31]. Another study in Tanzania showed that the concentrations of Mg, Mn, Fe, and Ni in medicinal plants were higher than the allowed limits [32]. It is important to emphasize that even if the heavy metal contamination is within the internationally accepted limits, there is still a high chance of accumulation of these elements in the body that reach toxic levels [33]. In the Middle East, the results of several studies showed that the heavy metals, such as Pb, Cd, Cu, and Zn in the herbal samples that were tested, were above the allowable limits. These results indicate that long-term use of contaminated herbal products may pose a potential health risk to consumers [23, 34, 35]. A comparative assessment of the quality of commercial black and green tea was conducted in Italy to check microbial and fungal contamination (total bacterial count, fungi, *Escherichia coli*, *Pseudomonas* spp., *and Clostridium perfringens*). The study showed that there was no microbial contamination that could be harmful to consumers, except for the contamination by mycotoxins such as ochratoxin A, which was above the allowable limits in food products in 50% of analyzed samples [36].

The objective of our study was to perform a quality check for the microbial and heavy metal contamination in green tea and herbal tea that claims to aid weight loss and is widely used among the Palestinian population. The results of the study reflected the actual situation of this problem in Palestine and will help raise the issue of contamination in herbal products to the concerned authorities.

## 2. Materials and Methods

### 2.1. Chemicals and Reagents

The following reagents were used throughout this study: tryptic soy agar (Difco Laboratories, Detroit, USA), Sabouraud dextrose agar (Difco™; France) (for preparing the medium), tryptic soy broth (Difco™; France), and Tween 80 (Difco™; France), which was used to prepare Fluid 3 for microbiological testing. Nitric acid (70%) (Riedel-de Haen, Seelze, Germany) and perchloric acid (70%–72%) (Riedel-de Haen, Seelze, Germany) were used for acid digestion of the herbal samples.

### 2.2. Instrumentation

An atomic absorption spectrometer (model: iCE 3000; Thermo Fisher Scientific) was used for the quantitative analysis of heavy metals. An autoclave (model: DLOV 3764; De Lama) was used for the sterilization of Fluid 3 (F3) and the agar mediums. A laminar air flow chamber (BBS-V1300; Biobase) and incubators (BC3100-R1; Biorold) were used for microbiological testing of the herbal samples, and Vortex Genie 2 was used for sample mixing.

### 2.3. Collection of Tea Samples

Eighteen samples were collected from either herbal medicine shops or community pharmacies. The samples were either green tea or herbal tea used for weight loss belonging to widely used brands in Palestine. Each tea sample was from a different brand, and the samples were either packed locally in Palestine or imported. The collected tea samples were primarily and widely used for weight loss.

All the samples that were collected were fine particles; thus, the samples did not require any further processing before experimentation.

They were stored in airtight glass containers in a dark, dry, and cool place during the period of the study.

### 2.4. Elemental Analysis

#### 2.4.1. Acid Digestion of the Herbal Sample

The acid digest was prepared by oxidizing 0.2 g of the dry powder from tea bags, with 10 mL of an acid mixture of 2 : 1 ratio (nitric acid: perchloric acid), which was then stirred and kept overnight at room temperature. The mixture was then filtered, and 1 mL of the filtrate was diluted to 25 mL with distilled water. The diluted samples were used in the quantitative measurement of Pb, Cd, Cu, Ni, Cr, and Zn, using an atomic absorption spectrophotometer. These samples were prepared in triplicate for each tea brand, and each of those were tested three times; the average values and standard deviations were recorded.

#### 2.4.2. Quantitative Elemental Analysis

For analysis, four standard concentrations of each heavy metal were prepared. The digested samples were diluted with water to be within the range of the calibration curve. The concentrations of the heavy metals in the samples were determined using the calibration curve and regression equation [37]. The four-point calibration of the concentration for each heavy metal that was studied is provided in Table 2.

### 2.5. Microbiological Testing

The tests for bacterial and fungal contamination were conducted according to the United States Pharmacopoeia (USP) [38]. Fluid 3 “F3,” a medium which was prepared by adding 15 g tryptic soy broth to 500 mL distilled water and 1 mL of Tween 80, was used to test for bacterial growth. Another medium was prepared by suspending 40 g of the powder of tryptic soy agar in 1 L of purified water. The mixture was thoroughly stirred with heat and boiled for 1 min to dissolve the powder completely.

Sabouraud dextrose agar was used to test for yeast and fungal growth. It was prepared by dissolving 65 g of the powder in 1 L of purified water, mixed thoroughly with heat, and frequently agitating to dissolve the powder completely. The agar medium was autoclaved at 121°C for 15 min. This medium was then used for the inoculation of fungi. The medium was placed in an oven at 50°C to maintain the liquid phase and was used whenever required.

Consequently, dry tea samples (3 g) were added to the corresponding preparations of Fluid 3 (30 g). The samples were diluted to prepare the following concentrations: 10, 10^2^, 10^3^, and 10^4^. Each dilution was inoculated on plates of tryptic soy agar and were incubated at 30–35°C for 48 h for bacterial identification. Fungal testing was performed by inoculating the prepared dilutions on plates of Sabouraud dextrose agar and incubating the plates at 20–25°C for 5 days. The results were reported as colony-forming units (CFU) per gram or mL.

### 2.6. Statistical Analysis

The Statistical Package for the Social Sciences software (SPSS) was used to perform descriptive statistical analysis. Analysis of variance (ANOVA) was used to test if there was a statistically significant difference between the average values. Pearson's chi-squared test was used to determine whether there was a significant difference between the expected and observed frequencies of more than one category. In all the statistical tests, if the *p* value was less than 0.05, the null hypothesis was rejected, and it was concluded that a significant difference exists.

## 3. Results and Discussion

**Figure 1:**
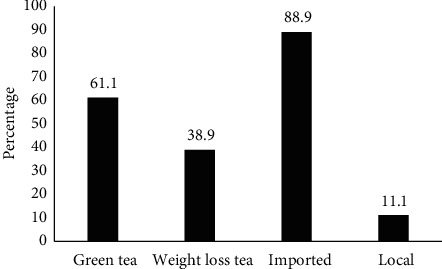
Source of tea samples.

Products for weight loss that are made from natural sources are widely used by consumers because of their belief in its safety. Green tea is one of the most common natural ingredients in over-the-counter products for weight loss, which are available worldwide. A total of 18 samples of different brands of tea were analyzed for heavy metal and microbial contamination. The samples were either of green tea or herbal tea, which is frequently used by people who follow weight loss regimens. The majority of the tested samples were of green tea (60%), while other herbal teas constituted 39% of the tested samples. These percentages reflect the consumption by the public because a majority of the people think that green tea is safer as well as cheaper than other herbal teas for weight loss (Figure 1).

The tea samples were either locally packed or imported from other countries. Majority of the samples were imported from other countries (89 %), while the rest (11 %) were from local sources (Figure 1). The source of tea sample reflects the trends in selling herbal products in the local Palestinian market. The majority of herbal products, which are sold as weight loss products, are imported. There are only few local manufacturers of herbal medicine in Palestine; thus, their share in the local market is low compared to the imported products [39].

The results of the test for heavy metal contamination in the samples showed that all the samples had passed the test for Cu, Ni, and Cd and failed the test for Cr. Additionally, 3 of the samples had failed the test for Pb. The concentration of each heavy metal that was identified in each sample are provided in Table 3.

**Figure 2:**
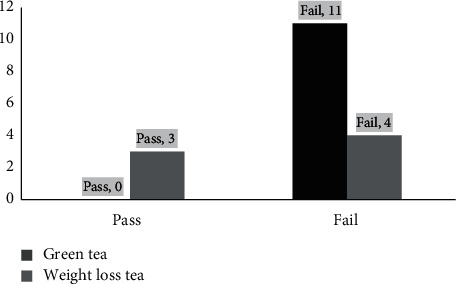
Concentration of lead in both types of tea samples.

The resulting concentration of Pb varied depending on the type of tea sample. Notably, all green tea samples had passed the test for Pb, while only 4 out of 7 samples of herbal tea had passed this test (Figure 2). Therefore, we performed a chi-squared test to determine the statistical difference, and the result showed significant difference (*p* = 0.043) between green tea and herbal tea. These results show that the lead contamination is less in green tea compared to herbal tea. Herbal products for weight loss consist of a mixture of many herbs that are cultivated from many sources; therefore, some of the content may contribute to high lead levels [40]. Moreover, most of the herbal products for weight loss that are present in the Palestinian local market are not registered; thus, these items are not continuously monitored, and many of them could be adulterated and counterfeit brands [41].

**Figure 3:**
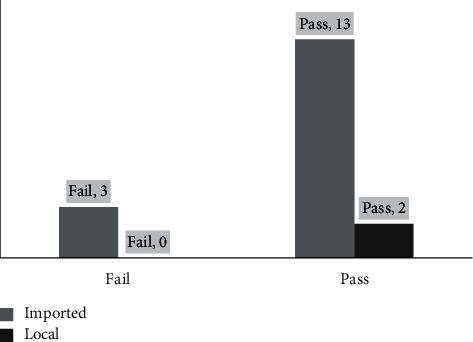
Concentrations of lead in the samples based on the source.

Further statistical analysis was performed to determine whether the different source of the tea samples attributed to the lead contamination (Figure 3). A chi-squared test was performed to determine if the difference was statistically significant. The results clearly showed that there was no significance (*p* = 0.66). This indicates the quality of both types of teas, regardless of the source. As mentioned earlier, imported herbal products are not registered under the MoH, and many of them are contaminated due to improper handling, storage, and packing in local Palestinian establishments [42].

The results of the microbial tests showed that 33 % of the samples had microbial contamination.

**Figure 4:**
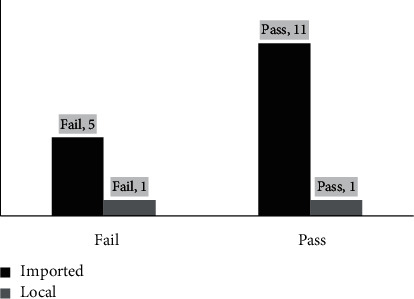
Results of microbial tests of the samples based on the source.

The results of microbial tests from both sources of tea samples are shown in Figure 4. Six out of 18 samples had failed the test; 4 of the samples had a TYMC above the allowable limit (100 CFU/mL), and 1 sample had a TAMC above the allowable limit (1000 CFU/mL) [43, 44]. Moreover, 1 of the sample was contaminated with *E. coli*. The chi-squared test showed no significant difference in contamination between the imported and the local sources of tea samples (*p* = 0.59).

**Figure 5:**
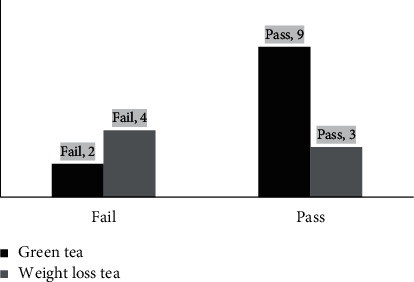
Results of microbial tests of both types of teas.

The results of the microbial tests of both types of teas are shown in Figure 5. The results showed that 2 out of the 11 green tea samples had failed the microbial test, and 4 out of the 7 samples were found to have microbial contamination. The results clearly show that the herbal tea was more contaminated than green tea. This could be because most of the brands of herbal tea for weight loss were not registered under the MoH; thus, they were not tested and did not undergo auditing. However, the chi-squared test showed no statistical significance (*p* = 0.12). Microbial contamination in herbal products could be attributed to improper handling during dispensing, packaging, and/or nonadherence to good manufacturing practices [45, 46].

## 4. Conclusion

Green tea and weight loss herbal tea are widely used in the local and international market. Contaminated herbal tea proved to have detrimental and health hazards to its users specially when taken in large amount such as this types of widely used tea. This is the first study that evaluated the heavy metal and microbial contamination in green tea and herbal tea that are widely used for weight loss in Palestine. The results showed that many of the tested samples contain toxic heavy metals such as Cr and Pb, which were above the allowable limits. Moreover, majority of the tested samples had microbial contamination with *E. coli*, and some of the samples had TAMC and TYMC that were above the allowable limits. This study is a forewarning to all the responsible authorities, including the MoH, to take immediate corrective actions, such as quality control testing, auditing for proper storage conditions, and registration of marketed tea products.

## Figures and Tables

**Table 1:** Examples of allowable limits of toxic heavy metals according to the World Health Organization (WHO).

Element	For edible plants (mg/kg)	For medicinal plants (mg/kg)
Nickel (Ni)	1.63	1.5
Copper (Cu)	3	10
Zinc (Zn)	27.4	50
Cadmium (Cd)	0.2	0.3
Lead (Pb)	0.43	10
Chromium (Cr)	0.02	1.3

**Table 2:** Calibration of concentration for each heavy metal.

Metals	Ni (ppm)	Cu (ppm)	Zn (ppm)	Cd (ppm)	Pb (ppm)	Cr (ppm)
Standard #1	0.05	0.5	0.5	0.5	1	0.5
Standard #2	0.1	1	1	1	2.5	1
Standard #3	0.5	2	2	2	5	2
Standard #4	1	3	3	3	10	3

**Table 3:** Concentration (ppm) of each heavy metal that was identified.

No.	Lead (Pb)		Nickel (Ni)		Chrome (Cr)		Zinc (Zn)		Copper (Cu)		Cadmium (Cd)	
1	0.101 ± 0.0103	Pass	0.248 ± 0.0270	Pass	0.234 ± 0.0340	Fail	0.063 ± 0.0088	Pass	0.029 ± 0.0210	Pass	0.011 ± 0.0109	Pass
2	0.110 ± 0.0303	Pass	0.355 ± 0.0151	Pass	0.251 ± 0.0460	Fail	0.074 ± 0.002	Pass	0.042 ± 0.0359	Pass	0.012 ± 0.0015	Pass
3	0.111 ± 0.0101	Pass	0.363 ± 0.0010	Pass	0.295 ± 0.0247	Fail	0.118 ± 0.010	Pass	0.534 ± 0.8439	Pass	0.012 ± 0.0019	Pass
4	0.122 ± 0.0303	Pass	0.421 ± 0.0242	Pass	0.345 ± 0.0223	Fail	0.121 ± 0.0087	Pass	0.019 ± 0.0233	Pass	0.014 ± 0.0025	Pass
5	0.123 ± 0.0030	Pass	0.449 ± 0.0283	Pass	0.386 ± 0.0280	Fail	0.124 ± 0.0171	Pass	0.014 ± 0.0219	Pass	0.011 ± 0.0008	Pass
6	0.112 ± 0.0122	Pass	0.455 ± 0.0480	Pass	0.424 ± 0.0170	Fail	0.156 ± 0.0482	Pass	0.018 ± 0.0171	Pass	0.018 ± 0.0006	Pass
7	0.110 ± 0.0123	Pass	0.514 ± 0.0359	Pass	0.462 ± 0.003	Fail	0.111 ± 0.0011	Pass	0.033 ± 0.0241	Pass	0.019 ± 0.0023	Pass
8	0.134 ± 0.0332	Pass	0.487 ± 0.0034	Pass	0.473 ± 0.0156	Fail	0.117 ± 0.0050	Pass	0.033 ± 0.0297	Pass	0.028 ± 0.0026	Pass
9	0.123 ± 0.0112	Pass	0.539 ± 0.0342	Pass	0.495 ± 0.0073	Fail	0.159 ± 0.0567	Pass	0.027 ± 0.0332	Pass	0.021 ± 0.0041	Pass
10	0.110 ± 0.0101	Pass	0.564 ± 0.0270	Pass	0.522 ± 0.0055	Fail	0.137 ± 0.0134	Pass	0.039 ± 0.0364	Pass	0.019 ± 0.0021	Pass
11	0.385 ± 0.1104	Pass	0.555 ± 0.0409	Pass	0.505 ± 0.0131	Fail	0.134 ± 0.0042	Pass	0.034 ± 0.020	Pass	0.031 ± 0.0020	Pass
12	0.449 ± 0.0397	Fail	0.586 ± 0.0138	Pass	0.564 ± 0.0143	Fail	0.125 ± 0.0057	Pass	0.021 ± 0.0203	Pass	0.031 ± 0.0020	Pass
13	0.381 ± 0.0460	Pass	0.571 ± 0.0315	Pass	0.539 ± 0.0092	Fail	0.128 ± 0.0039	Pass	0.051 ± 0.0498	Pass	0.039 ± 0.0042	Pass
14	0.374 ± 0.0377	Pass	0.579 ± 0.0123	Pass	0.640 ± 0.0022	Fail	0.171 ± 0.0152	Pass	0.121 ± 0.0252	Pass	0.041 ± 0.0027	Pass
15	0.369 ± 0.0234	Pass	0.562 ± 0.032	Pass	0.560 ± 0.0188	Fail	0.132 ± 0.0072	Pass	0.037 ± 0.0370	Pass	0.045 ± 0.0059	Pass
16	0.476 ± 0.0403	Fail	0.623 ± 0.0107	Pass	0.614 ± 0.049	Fail	0.139 ± 0.0113	Pass	0.013 ± 0.0140	Pass	0.050 ± 0.0031	Pass
17	0.484 ± 0.0911	Fail	0.595 ± 0.0078	Pass	0.646 ± 0.0260	Fail	0.126 ± 0.0034	Pass	0.020 ± 0.0285	Pass	0.050 ± 0.0012	Pass
18	0.340 ± 0.0303	Pass	0.614 ± 0.0232	Pass	0.640 ± 0.0113	Fail	0.132 ± 0.0016	Pass	0.034 ± 0.0286	Pass	0.0473 ± 0.0044	Pass

## Data Availability

No data were used to support this study
